# Impact of abiotic factors, habitat type and urban wildlife on the ecology of hard ticks (Acari: Ixodidae) in urban and peri-urban habitats

**DOI:** 10.1186/s13071-020-04352-3

**Published:** 2020-09-18

**Authors:** Silvia-Diana Borşan, Andra Toma-Naic, Áron Péter, Attila D. Sándor, Cosmin Peștean, Andrei-Daniel Mihalca

**Affiliations:** 1 Department of Parasitology and Parasitic Diseases, University of Agricultural Sciences and Veterinary Medicine of Cluj-Napoca, Cluj-Napoca, Romania; 2 Department of Parasitology and Zoology, University of Veterinary Medicine, Budapest, Hungary; 3 Department of Surgery, Anesthesiology and Intensive Therapy, University of Agricultural Sciences and Veterinary Medicine of Cluj-Napoca, Cluj-Napoca, Romania

**Keywords:** Ixodidae, *Ixodes ricinus*, *Haemaphysalis punctata*, Recreation areas, Urban wildlife

## Abstract

**Background:**

Ticks are increasingly acknowledged as significant vectors for a wide array of pathogens in urban environments with reports of abundant tick populations in recreational areas. The study aims to contribute to a better knowledge of the abiotic and biotic factors which impact the ecology of hard ticks in urban and peri-urban habitats in Romania.

**Methods:**

Questing ticks were collected by flagging in seven recreational locations, from four types of habitats in Cluj-Napoca, Romania: parks; gardens; a cemetery; and peri-urban forests. Hedgehogs, birds and micromammals were also sampled and searched for ticks, using standard methods (i.e. torch-based searches, ornithological mist nets, snap-traps, etc.), while vegetation was evaluated on surveyed areas. Data on questing ticks were converted to abundance indices. Moodʼs median tests were used to assess the relationship between the abiotic and biotic factors and the abundance of questing ticks.

**Results:**

Two species of questing ticks were found: *Ixodes ricinus* (96.8%) and *Haemaphysalis punctata* (3.2%). *Ixodes ricinus* was also the predominant engorged tick collected from urban wildlife. For *I. ricinus* the highest mean total abundance index/location (total no. of ticks/100 m^2^) was recorded in the urban gardens (3.79, 95% CI: ± 1.59) and parks (2.68, 95% CI: ± 0.75), whereas the lowest mean total abundance index was noted in the peri-urban forests (0.06, 95% CI: ± 0.03) and the urban cemetery (0.04, 95% CI: ± 0.02). The adults and nymphs of *I. ricinus* displayed a bimodal activity pattern, while the larvae showed a unimodal questing behaviour with an autumnal peak. Positive correlations were found between the mean total abundance index and the rise in the daily mean temperature and relative humidity, and between the global abundance of questing ticks and the presence of hedgehogs in the respective locations (*P* < 0.01).

**Conclusions:**

Ticks were collected in all the recreational sites surveyed in Cluj-Napoca. *Ixodes ricinus* was the dominant questing and engorged tick species. Several abiotic and biotic factors shape the ecology of ticks in Cluj-Napoca city, with climate and the local presence of suitable hosts being the most important.
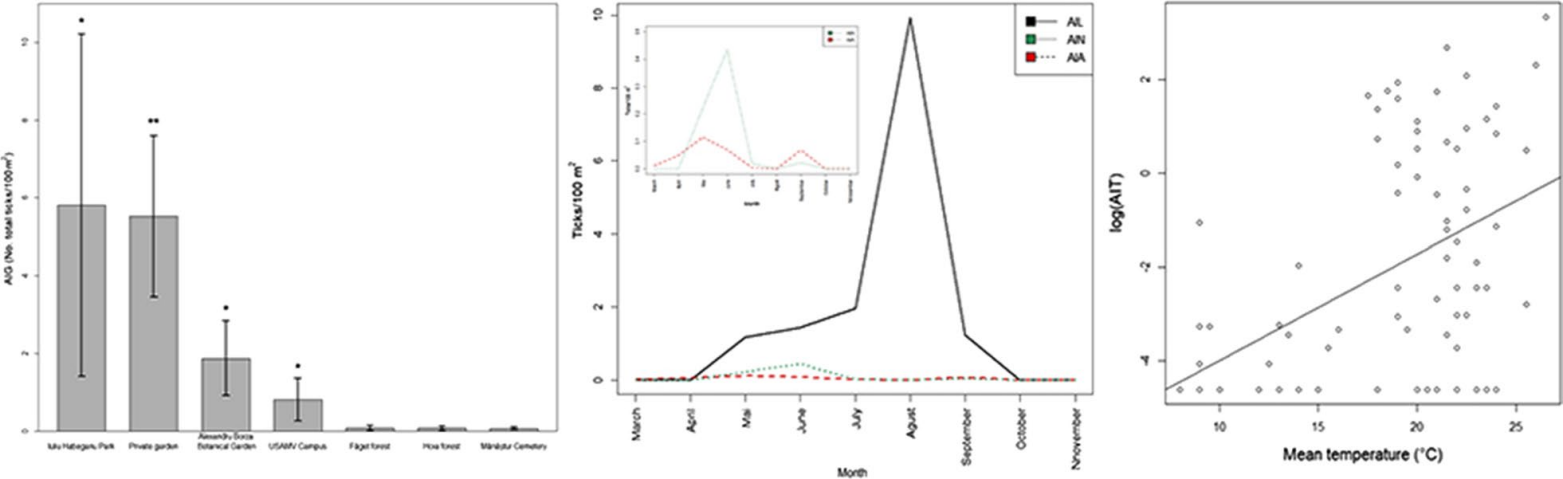

## Background

Considering their vectorial role, ticks are amongst the most important blood-feeding arthropods globally. Surpassed only by mosquitoes, ticks circulate a great diversity of pathogens which are known to cause many animal and human diseases worldwide [1, 2].

Several ecological factors can influence both the host and the tick populations, thus they shape the eco-epidemiology of zoonotic tick-borne diseases [3]. Climate and land-use changes and the recent increase in urbanization play a key role in the emergence of human tick-borne pathogens [4–6].

Ticks have long been included in the urban fauna, with numerous reports of their presence particularly in suburban areas [6]. The worldwide increase of the urbanisation rate in the recent decades has significantly altered biodiversity levels and impacted both tick-host assemblages and tick infection rates [5]. In Europe, the recent increase in peri-urbanisation and the human activities related to this transformation, depict urban and peri-urban sites as favourable habitats for human-tick encounters. The patterns of urbanisation are distinct in Europe, showing a network of cities denser than elsewere in the world [7]. In many European settlements, green areas inside cities such as forests, gardens, parks, cemeteries, and large private properties in peri-urban areas have been preseved as an effort to increase urban living standards. Such locations provide appropriate environmental conditions and sufficient host densities for the development of ticks, and maintainance of tick-borne pathogen foci [6, 8, 9].

Of the *c.*700 hard tick (Ixodidae) species described so far, the most relevant genera for public health are *Ixodes*, *Haemaphysalis*, *Dermacentor*, *Rhipicephalus*, *Hyalomma* and *Amblyomma* [10]. The generalist *I. ricinus* is Europe’s most prominent and widely distributed tick species and a vector for pathogens of public health relevance such as the tick-borne encephalitis virus, *Borrelia burgdorferi* (*s.l*.), *B. miyamotoi*, *Anaplasma phagocytophilum*, *Rickettsia helvetica*, *R. slovaca*, *R. monacensis*, *Babesia divergens*, *B. microti*, *B. venatorum* and *Bartonella henselae* [7, 9, 11]. Recent reports concerning *I. ricinus* underline several ecological changes such as an increase in abundance and questing periods and expansion of habitat including urbanized areas, all assigned to various interconnected factors including microclimate and climate parameters, habitat characteristics, or host availability [10, 12, 13]. Regarding the latter factor, it is worth mentioning that most urban wildlife species are highly adaptable due to their generalist behaviour and can reach even higher population densities than in natural sites [14]. The majority of urban-dwelling vertebrate species can act both as tick-maintenance hosts and reservoirs of tick-borne pathogens, thus playing an important role in the distribution and maintenance of tick populations and associated TBPs [10]. While *I. ricinus* is not the only tick species present in urbanised areas, there is a paucity of data regarding the distribution and vectorial capacity of other medically significant tick species in Europe’s urban and peri-urban sites [15, 16].

In Romania, major adjustments in land-use and abandonment rate, urbanization, and expansion of agriculture marked the outcomes of agricultural and industrial revolutions [17]. Given its geographical position and with a moderate continental climate Cluj stands out as a county with great biological diversity, expressed both at ecosystem and species level [17]. Recently, the level of urbanization in Romania, as in Cluj county, has been on a constant rise. Still, Romania is dealing with a dearth of information concerning the ecology of tick populations and the risk of acquiring tick-borne diseases in urban recreational areas. In Romania, the studies that also include ticks collected in urban areas focus either on the pathogen diversity in questing ticks [18, 19] or the prevalence of pathogens in engorged ticks, collected from animal hosts [20, 21]. Additionally, other papers briefly report data on ticks biting humans in Cluj and Sibiu counties [22–25]. Apart from the study of Pavel et al. [26] in four urban areas in Iași county, there is no information concerning the ecology of ticks in urban areas in Romania.

Poor data regarding the hard tick fauna and tick-maintenance wildlife hosts in urban areas in Romania have motivated this research. The study aims to contribute to a better knowledge of the ecological factors which impact the diversity, abundance, and seasonality of hard ticks, consequently acting as major hazard determinants of tick-borne diseases in urban environments.

## Methods

### Study area and design

Cluj county, comprising an area of 6,674 km^2^, is situated in the north-western part of Romania. According to the Cluj County Directorate of Statistics [27] in 2019, the county of Cluj had a stable population of 732,267 inhabitants. Of the total population, 66% live in urban areas, while 34% of the inhabitants live in rural areas [17]. The majority of the urban population (321,687 inhabitants) lives in the municipality of Cluj-Napoca (46.76°N, 23.58°E) the largest city of the county, and the fourth most populous city in Romania in 2016 [28].

From March to November 2018, flagging, sampling and trapping campaigns were performed in five urban and two peri-urban locations inside Cluj-Napoca, Romania, as follows: the campus of the University of Agricultural Sciences and Veterinary Medicine (referred to as USAMV Campus); Mănăștur Cemetery; Iuliu Haţieganu Park; Alexandru Borza Botanical Garden; a private backyard garden (situated in the city centre); and Hoia and Făget peri-urban forests (Table 1). All locations, except for the private garden, are open to the public and used for leisure time activities. The private garden was included to assess the risk of human-tick encounters in a more restricted area.

**Table 1:** Characteristics of collection sites in Cluj-Napoca

Site no	Location	Geographical coordinates	Flagged surface (m^2^)	Habitat	Groups exposed to contact with ticks
1	USAMV Campus	46°45′39.7″N, 23°34′14.9″E	7094.11	University campus, also used as a city park with open grassy areas, areas with trees, bushes, hedges, leaf litter and ivy, the presence of a river and riparian vegetation	Students, academic and maintenance staff, nearby residents, visitors, tourists, pedestrians, children
2	Mănăștur Cemetery	46°45′22.1″N, 23°34′12.1″E	3890.55	Centrally located cemetery with small green belts and connective land with grass between graves, a few clearings bordered by hedges and sparse trees	Maintenance staff, residents, visitors
3	Iuliu Hațieganu Park	46°45′56.1″N, 23°33′42.9″E	3162.04	Centrally located park with open grassy areas, trees, flower beds, shrub, hedges and a riverside area with tall trees, leaflitter, ivy and unmanaged vegetation	Maintenance staff, nearby residents, visitors, tourists, children, pedestrians, during summer sunbathers, people who perform outdoor activities (dog walking, picnic, sports)
4	Alexandru Borza Botanical Garden	46°45′45.4″N, 23°35′18.8″E	3844.08	Botanical Garden situated in the city centre with exotic and autochthonous species of plants, grassy green areas, small mixed forests, shrub, bushes, ivy	Maintenance staff, residents, visitors, tourists, students, children
5	Hoia forest	46°46′44.6″N, 23°31′15.5″E	5109.23	Peri-urban deciduous forest with grassy meadows dominated by beach, hornbeam and pedunculate oak	Nearby residents, during summer sunbathers and people who perform outdoor activities in this area (dog walking, picnic, sports)
6	Făget forest	46°42′46.8″N, 23°32′48.1″E	13534.81	Peri-urban deciduous forest dominated by beech, hornbeam, and spruce with grassy meadows	Nearby residents, during summer sunbathers and people who perform outdoor activities in this area (dog walking, picnic, sports)
7	Private central garden	46°46′40.0″N, 23°34′54.5″E	296.90	Central backyard garden composed of grassy meadow with cherry trees, shrub, ivy and a vegetable garden	Owners (family and friends) while gardening or during leisure time activities

### Flagging

**Fig. 1:**
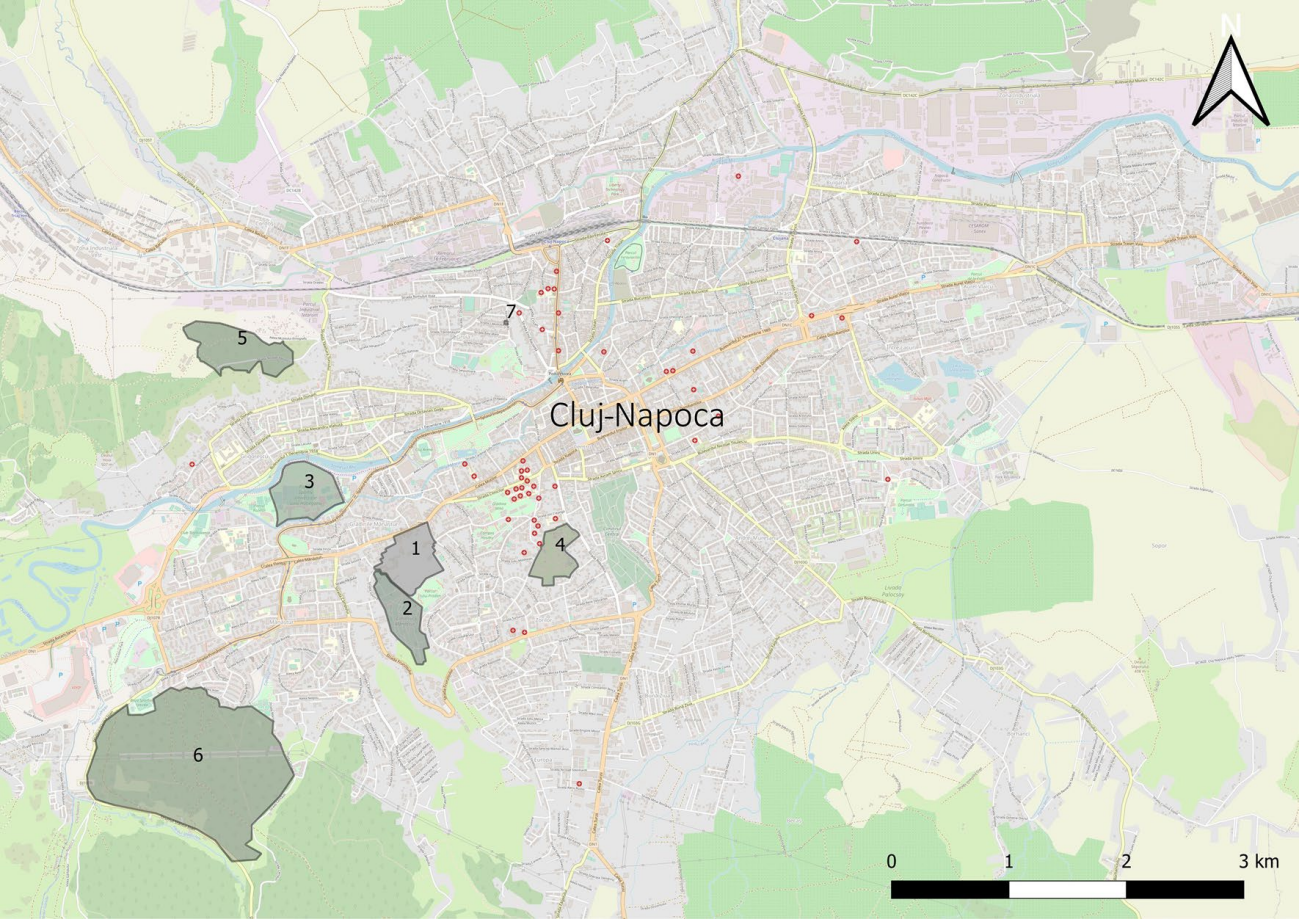
The geographical positioning of the collection sites. 1: USAMV Campus; 2: Mănăștur Cemetery; 3: Iuliu Haţieganu Park; 4: Alexandru Borza Botanical Garden; 5: Hoia forest; 6: Făget forest; 7: Private central garden

The flagging campaigns started in March 2018 and continued throughout the year, once every two months, until November, which marked the last successful sampling (defined as the third consecutive unsuccessful flagging event in all locations). For this particular activity (as well as for the micromammal trapping) the locations were divided into three groups (Fig. 1) according to their geographical position, as follows: Group 1 (USAMV Campus and Mănăştur Cemetery); Group 2 (Iuliu Haţieganu Park and Alexandru Borza Botanical Garden); and Group 3 (Hoia forest, Făget forest, and the private garden). Therefore, two or three locations were simultaneously assessed each week of the campaign for three consecutive days during the daytime between 9:00 h and 16:00 h. (Additional file 1: Table S1). The duration of each flagging event was 1 h per site when two experienced researchers simultaneously performed the flagging.

Some field visits were postponed for 1–3 days, due to unfavourable meteorological conditions. The areas surrounding natural or paved pathways, picnic areas, and footpaths that are usually frequented by people were of particular interest for each location. The flags consisted of flannel fabric (50 × 50 cm) attached to a wooden stick. All the collected ticks were stored in tubes with absolute ethanol. Standard variables (temperature: minimum, maximum, average; and relative humidity), were retrieved off-site for the locations on each date of sampling using the meteoblue meteorological archive graphics available online [29]. Because the standard variables were collected online, slight differences between our data (temperature and relative humidity) and the variables of the local microhabitats in each location are possible.

### Vegetation survey

The habitat type was recorded in each location (park, woodland, woodland edge, grassland and hedge) and the vegetation cover was assessed in each survey plot, using visual estimation of shrub, arboreal and perennial grass cover. Percentage cover of perennial grass and sward height (computed into 3 index categories: 0–20, 21–40 and > 41 cm height, measured using a ruler) was measured for each 100 m^2^ area where flagging was performed. Any bare area (pavement and gravel-covered walkways) were excluded. In addition we estimated the area of shrub layer (considered only where no grass was present below shrubs), while any area covered by closed canopy trees was assigned into arboreal cover in each plot. We computed a composite index (determined by log-transforming the % cover of the three major habitat types) for each surveyed area. Sward height (although measured) was discarded as regular grass management kept grass height below 20 cm in most areas, while grassy patches were scarce inside forests. In forests, major forest type was assigned into dominant if dominant tree species covered > 45%.

### Urban wildlife sampling

The purpose of this activity was to evaluate the role of birds, hedgehogs, and other rodents in the eco-epidemiology of ticks in urban environments. Host presence was monitored in each location using either standardised methods like torch-based searches (hedgehogs), ornithological mist nets (birds), snap-traps (micromammals), or through actual visualisation of hosts (domestic animals) or evidence of hosts (i.e.: faeces and birdsong).

#### Hedgehog sampling

The locations (all but the private garden) were screened for the presence of hedgehogs (see Additional file 1: Table S1). The searches were performed during the night (22:00–3:00 h) by two persons. The equipment consisted of headlamps and thick welding gloves for hedgehog handling. The caught hedgehogs were kept overnight, with animals being screened for ticks the following morning. Tick screening was performed under anaesthesia (performed at the Department of Surgery, Anesthesiology and Intensive Therapy of the Faculty of Veterinary Medicine of Cluj-Napoca by trained professionals to minimize the stress of the hedgehogs) to facilitate a thorough examination for the presence of ticks. Food was restricted until after anaesthesia, but water was available throughout the night before the procedure. The hedgehogs were anesthetized using the inhalation technique [30]. The induction was performed with isoflurane 5% in 100% oxygen administered into an induction chamber connected to a non-rebreathing system. The anaesthesia was also maintained using isoflurane 2% in oxygen delivered through a mask held over the animal’s nose [31]. After anaesthesia, all the hedgehogs were identified to species, sexed and weighed. All ticks were removed and stored in absolute ethanol. The tip of a bunch of spikes in the dorsal region was painted with light-coloured nail polish, for identification in case of recapture. The hedgehogs were monitored until full recovery from the anaesthesia. Before release, wet cat food and water were provided and during the same evening, all the hedgehogs were released back to the capture area.

#### Bird sampling

For this activity, three locations (USAMV Campus, Iuliu Haţieganu Park and Alexandru Borza Botanical Garden) were processed individually. The dates of the bird samplings are described in Additional file 1: Table S1. The campaigns lasted two consecutive days for each location. Three 12 m long ornithological mist nets with 5 longitudinal pockets were placed in various areas, where bird activity was previously noticed. The trapped birds were identified to species, age and sex [32]. Ticks, if present, were removed and collected in tubes with absolute ethanol. Before release, each bird was individually marked using standard metal rings.

#### Micromammal trapping

Micromammal trapping campaigns were conducted in all seven locations during the same days as the flagging activity (Additional file 1: Table S1). Seventy-five baited (peanut butter) snap-traps were placed in each location. The traps were positioned near dumpsters, alongside fences, hedges, under trees and bushes, along riverbeds, camouflaged in the vegetation, at an approximate distance of 1.5–2 m from one another. A coloured flag was placed by each trap, to facilitate the retrieving process. The traps were positioned on-site for three consecutive days. Daily check-ups were performed to collect the trapped micromammals and refill the traps with bait. All the micromammals were individually collected in transparent zip-bags. The identification of micromammals to species level was performed using morphological characters [33]. The micromammals underwent a thorough visual examination, with ticks being removed and stored in individual vials in absolute ethanol according to individual hosts.

### Tick identification

All ticks were labelled and stored at − 20 °C until further processing. Ticks were identified morphologically to species and developmental stage level. Species-specific identification was performed using the morphological keys in [34] under a stereomicroscope.

### Statistical analysis

To compare data between sampling sites, data on the questing tick species were converted to abundance indices (AI) which express the number of ticks collected per each 100 m^2^ flagged [35]. Due to the low numbers of other questing tick species, the AI was only calculated for *I. ricinus* ticks (separately for each stage, location and flagging event), as follows: the adults (males and females were considered together, due to the low number) (AIA); nymphs (AIN); larvae (AIL); and the total number of *I. ricinus* ticks per location (AIT). Another abundance parameter that was calculated for each location individually is the AIG. The AIG represents the global number of ticks collected in each location regardless of species and stage. Non-parametric Kruskall-Wallis tests and Moodʼs median tests were used to compare the AIG between locations. The conversion was performed according to the formula: AI = (TR × 100)/a, where TR represents the number of ticks collected, and a is the surface of the sampled area in square meters, as presented in [35]. Seasonal abundance values for *I. ricinus* ticks were tested in four locations in Cluj-Napoca (USAMV Campus, Iuliu Haţieganu Park, Alexandru Borza Botanical Garden and the private garden), using the abundance indexes AIL, AIN and AIA. The AIL, AIN, AIA and AIT of *I. ricinus* ticks were also used to determine the distribution of ticks concerning the mean temperature and relative humidity. Non-parametric correlation (Spearman’s rank correlation) was used to assess the relationship between climatic parameters (mean temperature and humidity) and AIT. The Wilcoxon rank-sum test was used to assess the importance of urban wildlife. The influence of the local vegetation on the abundance of questing ticks was tested by constructing generalised linear models (GLM from the “MASS”package) as described in [36]. The dependent variable was the tick abundance in each location (AIG) and the predictors were the recorded vegetation features (habitat and vegetation type and vegetation index). We also tested the habitat and vegetation type interaction, since different vegetation features can generate distinct effects within habitats. The initial model was reduced by backwards stepwise model selection, excluding the factor with the highest *P*-value. The statistical analysis was performed using R statistical software (3.4.3 version).

## Results

### Urban tick diversity

During March-November 2018, 3383 ticks were collected at the seven sampling sites (Table 2). The majority of the ticks were larvae (*n* = 3177), to a lesser extent, nymphs (*n* = 199) and adults (*n* = 136; 63 females and 73 males). The predominant species was *I. ricinus* (*n* = 3290; 97.2%) followed by *Haemaphysalis punctata* (*n* = 93; 2.7%). *Ixodes ricinus* was collected in all locations while *H. punctata* was present in all but two locations. Due to the low number of specimens, *H. punctata* was not included in further statistical analyses.

**Table 2:** Questing tick species collected in Cluj-Napoca

Species	Stages	USAMV Campus	Mănăştur Cemetery	Iuliu Haţieganu Park	Alexandru Borza Botanical Garden	Hoia forest	Făget forest	Private garden	Total
*I. ricinus*	Females	12	8	4	5	5	15	–	49
	Males	10	5	11	12	6	15	2	61
	Adults	22	13	15	17	11	30	2	110
	Nymphs	20	–	88	17	24	14	7	170
	Larvae	683	8	1542	621	5	4	147	3010
	Total	725	21	1645	655	40	48	156	3290
*H. punctata*	Females	1	9	2	–	–	1	–	13
	Males	2	8	2	–	–	3	–	15
	Adults	3	17	4	–	–	4	–	28
	Nymphs	2	3	7	–	1	22	–	35
	Larvae	–	1	–	–	6	23	–	30
	Total	5	21	11	–	7	49	–	93
Total		730	42	1656	655	47	97	156	3383

### The abundance of questing ticks

Significant differences were recorded in the AIT of *I. ricinus* ticks between the USAMV Campus and Iuliu Haţieganu Park (*Z* = 2.88, *P* < 0.001), between Iuliu Haţieganu Park and Hoia forest (*Z* = 3.09, *P* < 0.001), Făget forest (*Z* = 3.34, *P* < 0.001), Mănăştur Cemetery (*Z* = 2.96, *P* < 0.0075), and also between Alexandru Borza Botanical Garden and Hoia forest (*Z* = 3.92, *P* < 0.001), Făget forest (*Z* = 3.34, *P* < 0.001), Mănăştur Cemetery (*Z* = 4.17, *P* < 0.001). Also, a higher AIN was found at Iuliu Haţieganu Park (0.3) compared to USAMV Campus (0.01; *Z* =  − 3, *P* < 0.01) (Table 3).

**Table 3:** The AIL, AIN, AIA and AIT for *Ixodes ricinus*, and the AIG (all questing ticks regardless of tick species) in each location in Cluj-Napoca

Location	Abundance: mean (± 95% CI)				
	AIL	AIN	AIA	AIT	AIG
USAMV Campus	0.77 (± 0.56)	0.02 (± 0.02)	0.02 (± 0.03)	0.81 (± 0.53)	0.81 (± 0.55)
Mănăştur Cemetery	0.01 (± 0.01)	0.01 (± 0.01)	0.02 (± 0.02)	0.04 (± 0.02)	0.065 (± 0.03)
Iuliu Haţieganu Park	5.42 (± 4.52)	0.31 (± 0.38)	0.06 (± 0.16)	5.78 (± 4.41)	5.81 (± 4.4)
Alexandru Borza Botanical Garden	1.79 (± 1.46)	0.03 (± 0.03)	0.05 (± 0.04)	1.88 (± 0.96)	1.87 (± 0.96)
Hoia forest	0.02 (± 0.01)	0.03 (± 0.04)	0.02 (± 0.02)	0.06 (± 0.07)	0.07 (± 0.06)
Făget forest	0.01 (± 0.01)	0.02 (± 0.02)	0.02 (± 0.02)	0.04 (± 0.03)	0.073 (± 0.07)
Private garden	5.02 (± 2.17)	0.3 (± 0.23)	0.17 (± 0.6)	5.49 (± 2.08)	5.52 (± 2.07)

**Fig. 2:**
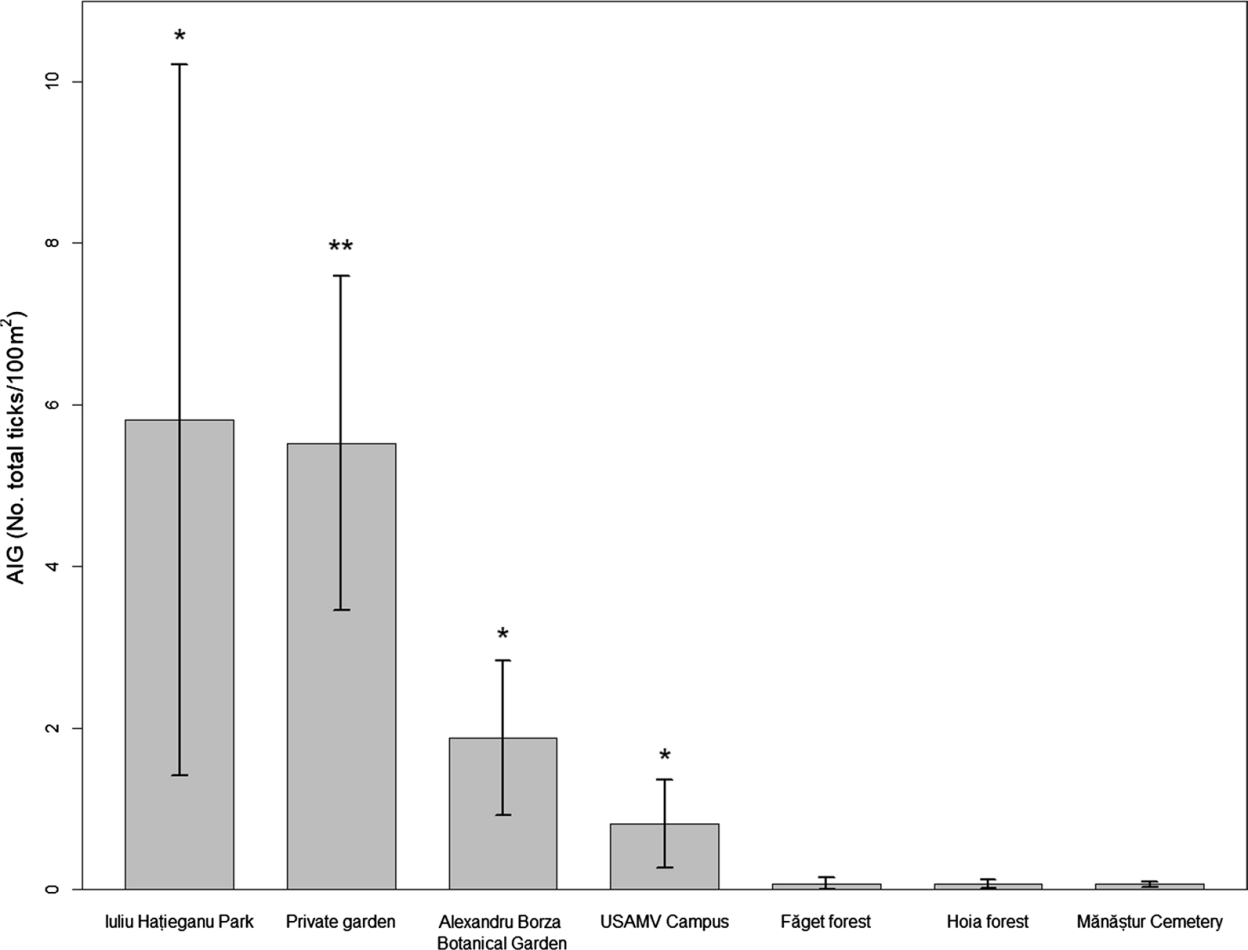
The AIG (all tick species together) of questing ticks in the seven locations in Cluj-Napoca. *Hedgehog presence confirmed; ** location not searched for hedgehogs

Due to the low AIL, AIN and AIA of Făget forest, Hoia forest and Mănăştur Cemetery, these locations were omitted from further comparison. The AI of *I. ricinus* ticks, considering all sampling sites together, were as follows: the AIL was 8.53/100 m^2^; the AIN was 0.47/100 m^2^; the AIA was 0.3/100 m^2^; and the AIT was 9.3/100 m^2^. The AIG differed significantly between locations (*χ*^2^ = 87.5, *df* = 55, *P* < 0.003) (Fig. 2). Variations in tick abundance between locations were compared using Moodʼs median test.

### Seasonality of questing ticks

**Fig. 3:**
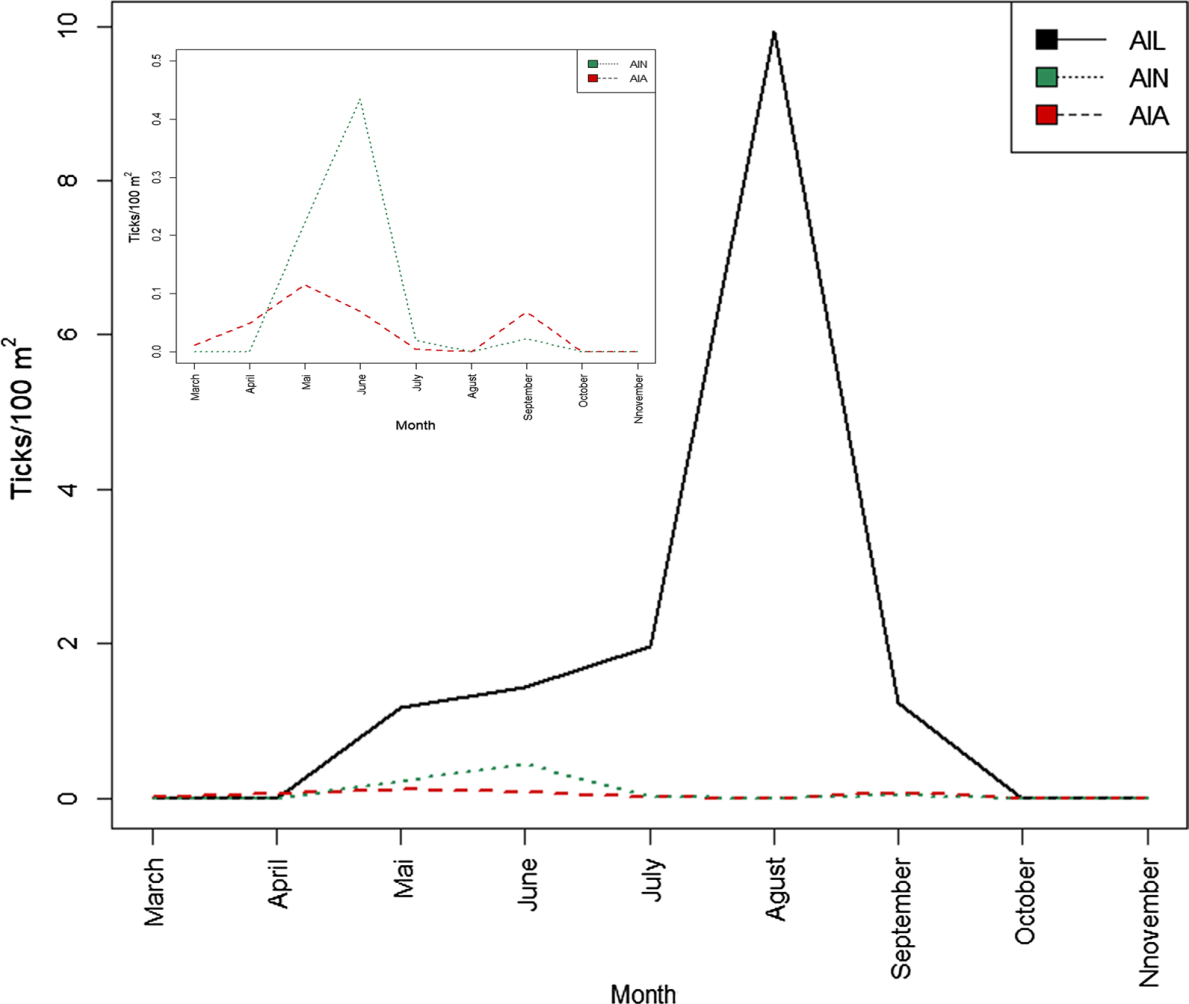
Seasonality of *I. ricinus* ticks in four recreational sites in Cluj-Napoca. The locations assessed are: USAMV Campus; Iuliu Hațieganu Park; Alexandru Borza Botanical Garden; and the private garden. For optimal viewing, a small scale graphic of the phenology of nymphs and adults is embedded in the top left corner of the image

Seasonal abundance values were evaluated in four locations (USAMV Campus, Iuliu Haţieganu Park, Alexandru Borza Botanical Garden and the private garden). The seasonal variation in the AI of each life stage of *I. ricinus* ticks is shown in (Fig. 3). The phenology of *I. ricinus* ticks in Cluj-Napoca showed a unimodal pattern of activity for larvae and a bimodal one for nymphs and adults. The first questing larvae were usually observed in May, and the highest AIL was detected between June and September, with a peak in August, followed by a lower number of larvae which remained active during autumn. Adults showed an early-onset of activity (March). Both the nymphs and the adults of *I. ricinus* were active during all three seasons, and presented a major spring to early summer peak (April-June), with a second, minor peak in September. Significant differences were found in USAMV Campus, concerning the AIL between spring (0.06) and summer (2.76; *P* < 0.05) and the AIA between spring (0.04) and autumn (0; *P* < 0.05). During spring, a higher AIL was observed at Alexandru Borza Botanical Garden (2.82) compared to USAMV Campus (0.1; *P* < 0.05). Also, during autumn a significantly higher AIN was found in Iuliu Haţieganu Park (0.13) compared to USAMV Campus (0; *P* < 0.01).

### The influence of abiotic factors on questing ticks

**Fig. 4:**
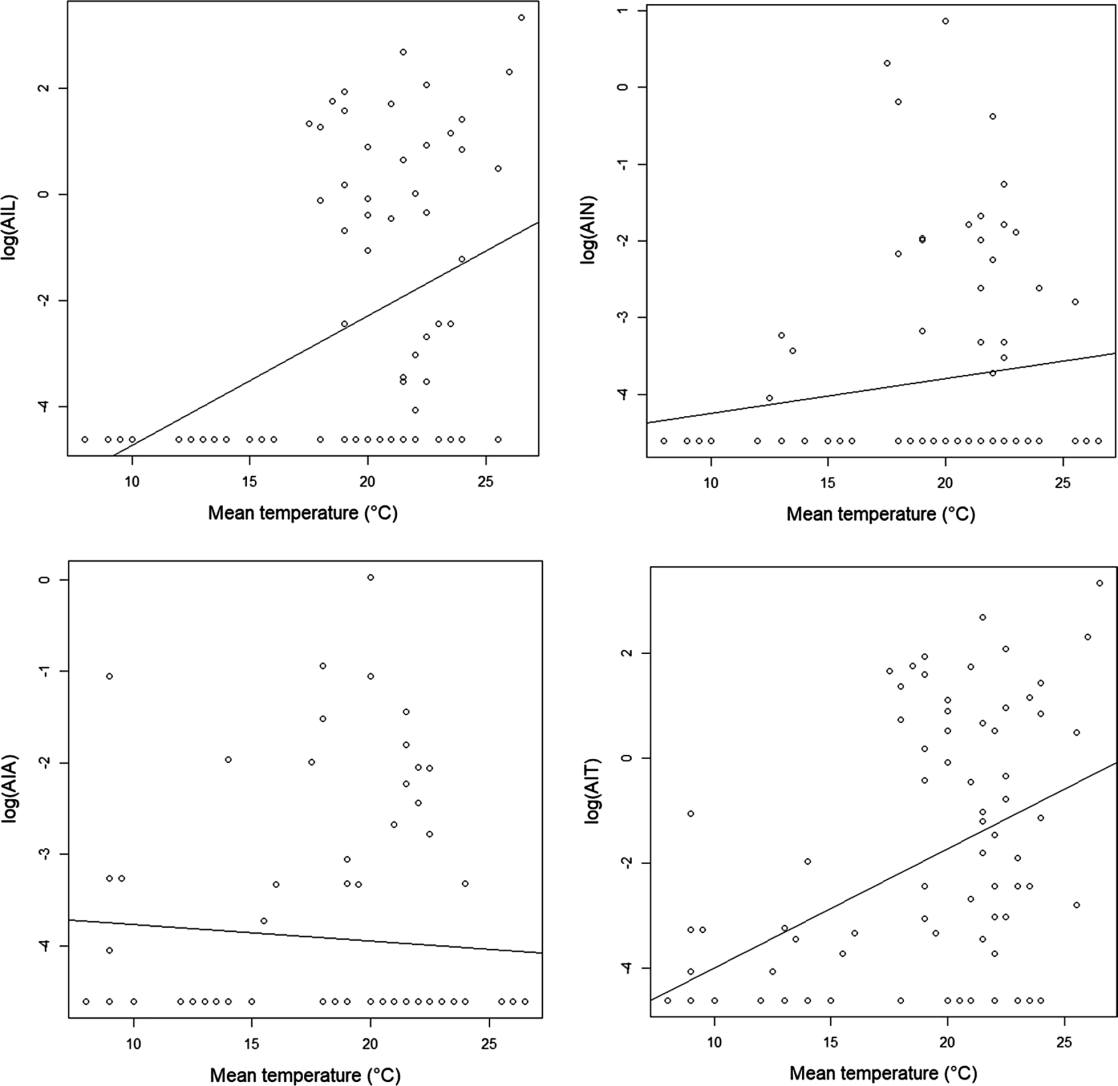
The abundance of *I. ricinus* ticks to mean temperature in Cluj-Napoca*. Abbreviations*: AIL, the abundance index of larvae; AIN, the abundance index of nymphs; AIA, the abundance index of adults; AIT, the total abundance of ticks

During the samplings, the average temperatures ranged between 2–33 °C. Larvae were present even at maximum daily temperatures above 30 °C, but no adults were found at 30 °C. Overall, in the examined range, there was a significant correlation between the abundance of questing ticks and the daily mean temperature of the flagging campaigns (Fig. 4). There was a significant positive correlation between the mean temperature, the AIT (rho = 0.369, *P* < 0.01), and the AIL (rho = 0.428, *P* < 0.01), while the AIA (correlation was marginally non-significant) showed a decrease in abundance with the increase of the mean temperature. Positive correlations were found between the maximum daily temperature and the AIL (rho = 0.353, *P* < 0.001) and the AIT (rho = 0.372, *P* < 0.001). Also, the AIL (rho = 0.527, *P* < 0.001) and the AIT (rho = 0.372, *P* < 0.001) correlated significantly with the rise of the minimum daily temperature. There was a weak negative correlation between the AIA (rho =  − 0.277, *P* = 0.005) and the minimum daily temperature.

**Fig. 5:**
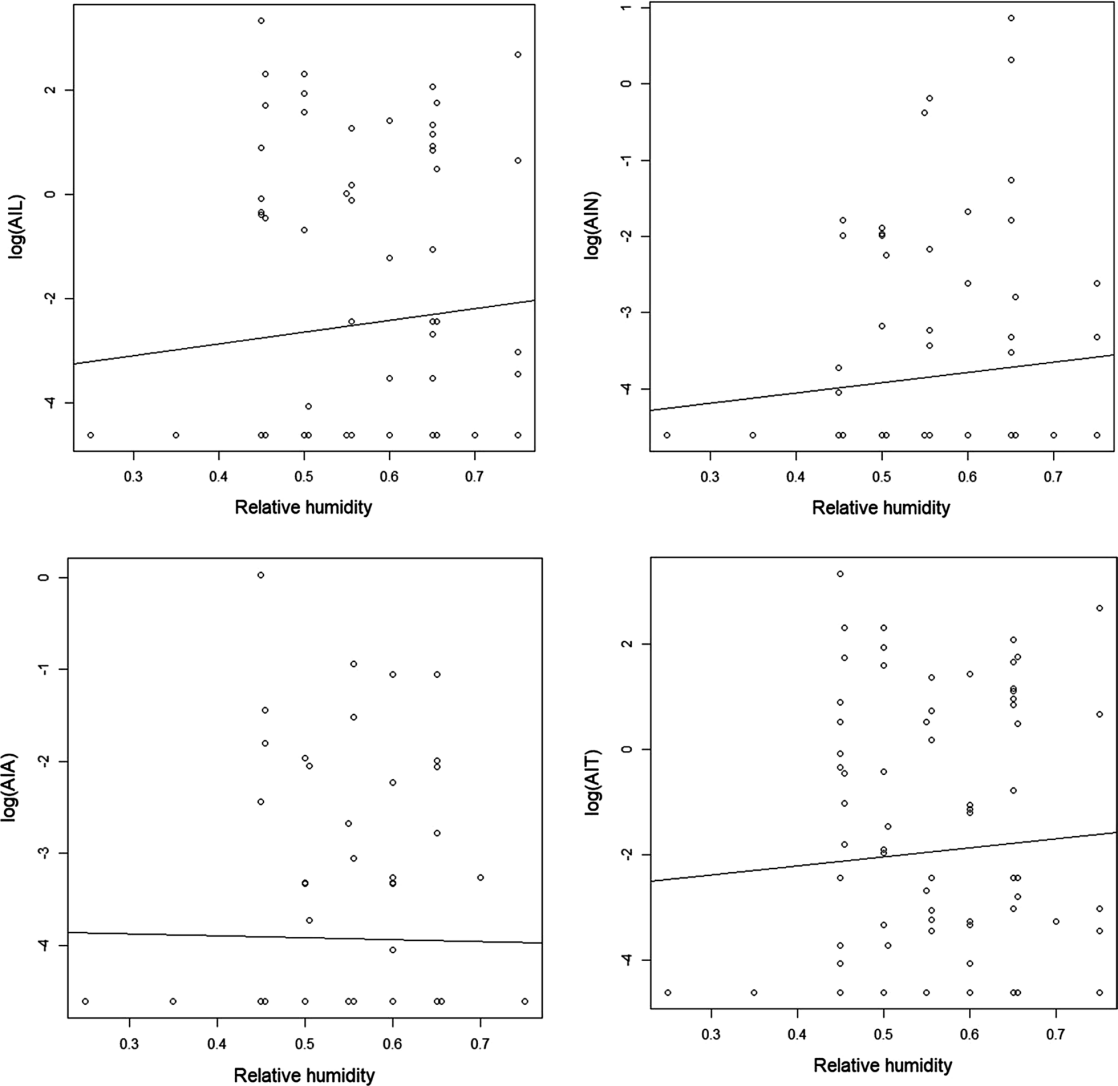
The abundance of *I. ricinus* ticks to the relative humidity in Cluj-Napoca*. Abbreviations*: AIL, the abundance index of larvae; AIN, the abundance index of nymphs; AIA, the abundance index of adults; AIT, the total abundance of ticks

During the samplings, the relative humidity ranged from 25 to 75%. An overall positive correlation was detected between the relative humidity and the AIL (rho = 0.218, *P* < 0.01; Fig. 5). Even so, a slight decrease can be noticed in the AIA (correlation was not significant) at a higher RH, probably due to the low number of adults compared to immature stages.

### Questing ticks and vegetation

The surveyed areas showed a diverse array of vegetation types, with grass/forb cover being the dominant (mean cover 69.22%, 95% CI: ± 0.053) and present in all sampled 10 × 10 m plots. Shrub layer was present in most plots, ranging from 10 to 60% cover (mean cover 17.55%, 95% CI: ± 0.035), while trees were dominant only in the two peri-urban forests (mean cover 22.75%, 95% CI: ± 0.052). There were major differences between individual locations in shrub or tree cover, while the differences in grass cover were not significant (Additional file 2: Table S2). We found no relationship between vegetation composition and tick abundance or distribution.

### Ticks on urban wildlife

Hedgehogs carried a considerably higher number of ticks (Table 4) than any other surveyed vertebrate species, therefore the data analysis regarding the influence of urban wildlife on the abundance of questing ticks was only performed for hedgehogs.

**Table 4:** Hard tick fauna of hedgehogs collected in Cluj-Napoca

Location	Host sex	*I. ricinus*					*I. hexagonus*					*H. punctata*					Total
		F	M	N	L	T	F	M	N	L	T	F	M	N	L	T	
USAMV Campus	♀	2	–	19	10	31	–	–	–	–	–	–	–	–	–	–	31
USAMV Campus	♂	22	8	129	8	167	–	–	–	–	–	–	–	3	–	–	170
USAMV Campus	♂	1	–	1	2	4	3	1	–	–	4	–	–	–	–	–	8
USAMV Campus	♂	1	–	9	22	32	4	–	–	–	4	–	–	–	–	–	36
Iuliu Hațieganu Park	♀	–	1	6	215	222	3	–	–	–	3	–	–	2	–	2	227
Iuliu Hațieganu Park	♀	–	–	3	32	35	–	–	–	1	1	–	–	1	1	2	38
Iuliu Hațieganu Park	♀	–	–	9	51	60	5	1	–	–	6	–	–	–	–	–	66
Iuliu Hațieganu Park	♀	–	2	18	287	307	2	1	–	–	3	–	–	2	19	21	331
Alexandru Borza Botanical Garden	♀	–	2	41	4	47	9	–	–	–	9	–	–	–	–	–	56
Alexandru Borza Botanical Garden	♀	9	5	6	8	28	–	–	–	–	–	–	–	–	–	–	28
Alexandru Borza Botanical Garden	♀	1	–	23	9	33	37	16	–	–	53	–	–	–	–	–	86

#### Hedgehogs

Eleven northern white-breasted hedgehogs (*Erinaceus roumanicus*) (8 females and 3 males), from Cluj-Napoca were screened for the presence of ticks. Hedgehogs were only found in three urban locations: USAMV Campus; Iuliu Hațieganu Park; and Alexandru Borza Botanical Garden. Engorged ticks were collected from all the captured hedgehogs. The number of ticks per hedgehog ranged from 8 to 331. Three of the 11 hedgehogs carried more than half of the ticks (728/1077). Tick burden did not significantly differ between sexes (*P* = 0.5) or locations (*χ*^2^ = 3.06, *df* = 2, *P* = 0.22). In total, 1077 ticks were identified to the species level using morphological keys (Table 4). *Ixodes ricinus* ticks were more prevalent (*n* = 966; 89.7%) followed by *I. hexagonus* (*n* = 83; 7.7%) and *H. punctata* (*n* = 28; 2.6%). The proportion based on the life stages for *I. ricinus* was as follows: larvae 67% (648/966); nymphs 27.3% (264/966); females 3.72% (36/966); and males 2.38% (23/966). For *I. hexagonus*, the identified stages were females 75.9% (63/83), males 22.9% (20/83), and larvae 1.2% (1/83). The proportion based on the life stage for *H. punctata* was: larvae 71.4% (20/28); and nymphs 28.5% (8/28); no adult was collected. Co-infestations (*n* = 9, 81.8%) were more common than infestation with only one species of tick (*n* = 2). Five hedgehogs were co-infested with *I. ricinus* and *I. hexagonus* ticks, 3 hedgehogs presented co-infestation with all three tick species, and 1 hedgehog was co-infested with *I. ricinus* and *H. punctata* ticks. The locations where hedgehog presence was confirmed had a higher average AIG (2.45) of questing ticks in comparison to the locations without hedgehogs (Wilcoxon rank-sum test, *P* < 0.01, Fig. 2).

#### Birds

A total of 57 birds were analysed during 2018 in Cluj-Napoca (Table 5). Of these, 11 (21.05%, 95% CI: ± 8.57) presented engorged ticks. From the 22 collected ticks, 68% (15/22; 12 nymphs and 3 larvae) were identified as *I. ricinus*, which was the most prevalent species feeding on the birds (*χ*^2^ = 7.6, *df* = 2, *P* = 0.02237). *Haemaphysalis* ticks were identified as *H. concinna* 22.7% (3 nymphs) and *H. punctata* 9% (2 larvae). The most common life stage retrieved from birds were nymphs (*n* = 17) followed by larvae (*n* = 5). Infestations with a single tick species (*n* = 7) were more common than co-infestations (*n* = 3). Ticks were collected from the following bird species: *Erithacus rubecula*; *Garrulus glandarius*; *Passer montanus*; *Phylloscopus collybita*; *Turdus merula*; and *Sturnus vulgaris* (Table 5). The most infested bird species was *S. vulgaris* with a prevalence of 50% and mean infestation intensity of 3 ticks (*n* = 2).

**Table 5:** Bird species and associated ticks collected in Cluj-Napoca

Host species	By location	With ticks	Ticks species and stage
*Columba livia* (*n* = 2)	USAMV Campus (*n* = 2)	–	–
*Corvus frugilegus* (*n* = 3)	USAMV Campus (*n* = 1); Iuliu Hațieganu Park (*n* = 2)	–	–
*Dendrocopos major* (*n* = 1)	USAMV Campus (*n* = 1)	–	–
*Erithacus rubecula* (*n* = 5)	USAMV Campus (*n* = 2); Iuliu Hațieganu Park (*n* = 1); Alexandru Borza Botanical Garden (*n* = 2)	USAMV Campus (*n* = 1)	1. IR × 1L + IR × 1 N
*Fringilla coelebs* (*n* = 2)	USAMV Campus (*n* = 2)	–	–
*Garrulus glandarius* (*n* = 1)	Iuliu Hațieganu Park (*n* = 1)	Iuliu Hațieganu Park (*n* = 1)	1. IR × 1L + IR × 1 N
*Parus major* (*n* = 13)	USAMV Campus (*n* = 4); Iuliu Hațieganu Park (*n* = 2); Alexandru Borza Botanical Garden (*n* = 7)	–	–
*Passer montanus* (*n* = 6)	USAMV Campus (*n* = 6)	USAMV Campus (*n* = 4)	IR × 1 N/each bird
*Phylloscopus collybita* (*n* = 3)	USAMV Campus (*n* = 3)	USAMV Campus (*n* = 1)	1. IR × 1 N
*Sitta europaea* (*n* = 2)	USAMV Campus (*n* = 2)	–	–
*Streptopelia decaocto* (*n* = 1)	USAMV Campus (*n* = 1)	–	–
*Sturnus vulgaris* (*n* = 4)	USAMV Campus (*n* = 4)	USAMV Campus (*n* = 2)	1. IR × 1 N + HC × 3 N; 2. HC × 2 N
*Sylvia atricapilla* (*n* = 1)	USAMV Campus (*n* = 1)	–	–
*Troglodytes troglodytes* (*n* = 2)	Alexandru Borza Botanical Garden (*n* = 2)	–	–
*Turdus merula* (*n* = 11)	USAMV Campus (*n* = 5); Alexandru Borza Botanical Garden (*n* = 6)	1. USAMV Campus (*n* = 1); 2. Alexandru Borza Botanical Garden (*n* = 1)	1. IR × 2 N; 2. IR × 1L + IR × 1 N + HP × 2L

*Abbreviations*: IR, *Ixodes ricinus*; HC, *Haemaphysalis concinna*; HP, *Haemaphysalis punctata*; L, larva; N, nymph

#### Micromammals

Altogether, 31 micromammals were collected and analysed during 2018 (Table 6). Eight (32%, 95% CI: ± 14) had one or multiple ticks attached. Ticks were collected from *Apodemus agrarius* (*n* = 3), *A. flavicollis* (*n* = 3), *Talpa europaea* (*n* = 1) and *Sorex minutus* (*n* = 1). The highest tick infestation was found in *A. flavicollis* (prevalence 50%, 95% CI: ± 38; mean intensity 3.6, 95% CI: ± 5.8). Twenty ticks were collected and identified morphologically as *I. ricinus* (1 nymph and 19 larvae). The majority of the ticks were found co-feeding on two *A. flavicollis* (*n* = 5 on each) and one *T. europaea* (*n* = 4). The ticks were clustered around the face, at the tip of the ears or on the tail. Except for the specified areas, ticks were not present on any other body parts.

**Table 6:** Micromammal species and associated ticks collected in Cluj-Napoca

Host species	By location	With ticks	Ticks species and stage
*Apodemus agrarius* (*n* = 14)	USAMV Campus (*n* = 7); Iuliu Hațieganu Park (*n* = 7)	Iuliu Hațieganu Park (*n* = 3)	1. IR × 1L; 2. IR × 1L; 3. IR × 2L
*Apodemus flavicollis* (*n* = 6)	USAMV Campus (*n* = 3); Alexandru Borza Botanical Garden (*n* = 1); Hoia forest (*n* = 2)	1. Alexandru Borza Botanical Garden (*n* = 1); 2. + 3. Hoia forest (*n* = 2)	1. IR × 5L; 2. IR × 5L; 3. IR × 1 N
*Apodemus sylvaticus* (*n* = 4)	USAMV Campus (*n* = 4)	-	-
*Mus musculus* (*n* = 1)	Hoia forest (*n* = 1)	-	-
*Muscardinus avellanarius* (*n* = 2)	Făget forest (*n* = 2)	-	-
*Myodes glareolus* (*n* = 1)	Hoia forest (*n* = 1)	-	-
*Sorex minutus* (*n* = 1)	Hoia forest (*n* = 1)	Hoia forest (*n* = 1)	1. IR × 1L
*Talpa europaea* (*n* = 1)	Alexandru Borza Botanical Garden (*n* = 1)	Alexandru Borza Botanical Garden (*n* = 1)	1. IR × 5L

*Abbreviations*: IR, *Ixodes ricinus*; L, larva; N, nymph

## Discussion

*Ixodes ricinus* is by far the most common hard tick in Europe’s [9] or Romania’s [37, 38] natural and urban habitats both regarding its distribution range and host spectrum. With two questing tick species, *I. ricinus* (*n* = 3290) and *H. punctata* (*n* = 93) collected in the seven locations in Cluj-Napoca, our results are comparable with the findings from other urban areas of Europe [36, 39, 40] or Romania [26]. Several studies in Cluj and neighbouring counties mentioned *I. ricinus* as the most abundant tick species feeding on humans [23, 25]. *Haemaphysalis punctata* was the only other questing tick in the urban and peri-urban sites in Cluj-Napoca. This species was present in five out of the seven flagged locations, with an overall prevalence of 2.7% (93/3383). Similar data were reported by [26] in north-eastern Romania. It is also the most common species of its genus parasitic in humans in Romania [23, 25, 41] and a vector for various *Rickettsia* spp., *Babesia* spp.[42], *Anaplasma* spp.[43].

Similar to data from Iași [26] and three regional parks in northern Italy [35], the AIL in this study was higher than the AIN and AIA. Generally, only 0.1% of the eggs produced by the female can reach the adult stage [44], therefore a lower abundance of adult stages compared to immatures is expected. Also, the collection of a greater number of larvae to nymphs or adults could be related to their highly aggregated distribution and lower dispersibility in the environment [45]. Still, an important shortcoming of our tick abundance analysis is the high number of larvae included in the study compared to the number of nymphs and adults.

In contrast with the findings of [46] we here report a significantly higher AIG of ticks in urban parks and gardens compared to the peri-urban forests. The GLM model used for the statistical analysis of vegetation and habitat predictors showed inconclusive results concerning local tick abundance in the seven sites. Even though the overall floral habitat composition did not influence tick abundance *per se*, the heterogeneity of these urban settlements is known to positively influence wildlife diversity [47]. Despite some variation of the ecological characteristics of urban green areas (*i.e.*: size, vegetation composition and level of human activity), USAMV Campus, Iuliu Haţieganu Park, and Alexandru Borza Botanical Garden are not the typical urban green spaces with highly manicured understories and intensive vegetation management. The three locations consist of a somewhat similar vegetation composition best described by mature native and exotic trees, some form of open water, the predominance of turfgrass and shrub understory and extensive areas of natural vegetation dominated by wooded parcels and abundant leaf litter, interspersed throughout the settlements or located at the periphery. The private garden consists of fruit trees, a vegetable garden, and was dominated by unmanaged turf and shrub understory (Table 1). These types of habitats provide excellent conditions for urban wildlife. Further studies are needed to assess the influence of microhabitat factors on tick abundance in urban and peri-urban areas, but in this case, the abundance of urban wildlife species, especially hedgehogs was statistically linked to a higher AIG of ticks in the locations where they were present. With few or no natural enemies, hedgehogs, songbirds, foxes, and squirrels, are some of the most successful urban dwellers [9]. The majority of these urbanised species, show a high tolerance to urban pressure, behavioural flexibility, well-adapted temperament trades [48], and are known as tick-maintenance and pathogen reservoir hosts [9]. Moreover, the urban hedgehogs supported all tick stages, thus proving that they can preserve stable *I. ricinus* populations in urban parks and gardens, even in the absence of larger hosts, such as roe deer [49]. First, compared to *E. europaeus*, [50] *E. roumanicus* was here found to carry more *I. ricinus* than *I. hexagonus* as already shown by Földvári et al. [51] in a city park of Budapest. Also in line with the latter study from the neighbouring Hungary, *E. roumanicus* was here also found to be an excellent maintenance host for *I. ricinus* in the absence of other local larger-sized mammal host populations. Although we found no correlations between the vegetation type or index, the low AIG of ticks in the peri-urban forests could be explained by a lower abundance and diversity of wildlife compared to the urban areas (Fig. 2; Table 6). Still, the high number of *H. punctata* ticks in Făget forest could be linked to the presence of a sheep stable close to the flagged meadows, which ensured appropriate hosts for this tick’s development [52]. The urban cemetery recorded the lowest AIG probably because of the lack of vegetated soil surface (high density of concrete memorial constructions, charnels, and concrete pavements restricting the surface of green belts and connective vegetation), while the surrounding concrete fence also limited the access of tick hosts, except birds [49]. Questing ticks were only found in small clearings surrounded by hedges or green belts, suggesting that ticks may be regularly introduced into the cemetery by birds from adjacent habitats [53], but a stable tick population is unlikely.

We observed that the highest AI of nymphal and adult questing ticks in Cluj-Napoca was in early summer (May–June) and early autumn (August–September), periods previously associated with a high risk of human-tick encounters in Cluj county [41]. This bimodal questing pattern of *I. ricinus* is common throughout Europe; nonetheless, major differences in the activity of tick stages can occur [54].

The bimodal questing activity described in highly seasonal climates, with early spring and autumn peaks of *I. ricinus* ticks [55] can also be observed for the *I. ricinus* nymphs and adults in Cluj-Napoca. Similarly to the urban areas in Iași, the larvae collected in Cluj-Napoca show a unimodal activity pattern with an autumnal peak, as previously reported [26]. A unimodal activity of ticks is typical for milder climates, with less climatic variation between seasons [55]. Nonetheless, the phenology of *I. ricinus* larvae in Cluj-Napoca can be either a result of the previous year (individuals overwintered as eggs or as larvae) or a product of eggs laid during the same spring [56]. The high larval peak in late summer, suggests that the latter is predominant in the current study area. Also, the co-existence of all three mobile life stages throughout the summer could enable the transmission of pathogens by co-feeding [57].

During sampling, we noticed a rise in AIL and AIT with increasing average temperature, in contrast to data obtained by Kubiak et al. [40] who reported reduced activity of ticks at high temperatures. Larvae are the most sensitive to high temperatures, which are usually associated with an increased mortality rate in *I. ricinus*, due to dehydration [58]. Boehnke et al. [59] concluded that information gathered by using official weather data (i.e. meteoblue) is not always a good proxy of the actual microclimatic conditions the ticks experience. Therefore, the increased AIL and AIT of ticks during the high temperatures recorded in this study, viewed as desiccating conditions, could be explained by the fact that the local microclimatic temperatures in the litter layer provided a continuous and sufficient moisture supply for questing ticks even during summer, despite the ambient factors recorded [59]. Since moisture is regarded as a crucial factor, especially for the optimal development of immature *I. ricinus* ticks, the correlation between the AIL and the increase of RH seen in this study is not unexpected [60].

Poor fauna is typical for the urban scenery [61]. Small to medium-sized mammals such as hedgehogs which can host all life stages of ticks [62] play an active part in the maintenance of TBPs in these areas. *Ixodes hexagonus* has been described in Romania from a variety of wildlife hosts [63, 64], and despite its endophilic behavior, sometimes it also bites humans [65]. In the present study, the number of hedgehogs with polyspecific infestations was the highest, and *I. hexagonus* was the second most prevalent tick species collected, after *I. ricinus*. Since both ixodid species can be found on hedgehogs and are known vectors for *B. burgdorferi* (*s.l*.) [50], *I. hexagonus* could contribute to the diffusion and persistence of pathogens in Cluj-Napoca by enzootic sub-cycles [50]. This is noteworthy for the urban and suburban environments where both hedgehogs and *I. ricinus* ticks reach high densities [10, 50].

Due to their foraging behaviour, birds also play an important role in the introduction of ticks and associated pathogens into urban sites [66–68]. The tick-infested urban birds in our study were mostly ground-feeders, either short-distance migratory species (*E. rubecula*, *S. vulgaris* and *T. merula*) or sedentary species (*G. glandarius* and *P. montanus*). Similarly to data provided by Sándor et al. [68] for southern Romania and Klaus et al. [69] for Germany, we report here higher tick infestation rates for short-distance migrants and sedentary birds, with a clear trend in ground- or shrub-feeding birds, rather than long-distance migrant or foliage gleaners. *Ixodes ricinus* was the dominant species collected from the urban birds in the present study, followed by *H. concinna* and *H. punctata*. Since urbanized birds seem to bear a higher number of ticks [69] some also vector for zoonotic pathogens [70], their importance as urban disseminators for the public health should not be neglected.

Our results showed a higher rate of micromammals trapped in the urban locations compared to the peri-urban sites in Cluj-Napoca (with a predominance of *A. agrarius* and *A. flavicollis*). This may be a result of a higher density of these species, caused by their increased winter survival rate and a longer breeding season in urban areas [71]. *Ixodes ricinus* was the dominant species feeding on urban micromammals too, similar to data reported by Maaz et al. [72] in urban areas in Berlin. Similarly to previous surveys in Romania [73] or in central Europe [74], we collected only larvae and nymphs from the trapped micromammals, thereby underlining the role of rodents as hosts especially for the subadult life stages of ticks not only in forested habitats but also in the urban sites [75].

Apart from the indisputable benefits of increasing urban green space and promoting its usage in developing cities [76], these areas can also serve as habitat for urban wildlife and their associated tick fauna [77]. Thus, cities should implement tick-management policies to control the risk of tick-borne diseases in urban premises. Maintenance practices such as mowing or shrubbery management should precede or coincide with the peak activity of questing *I. ricinus* nymphs and adults (March-July), while complementary approaches should include host-targeted methods which are preferred due to the reduced risk of acaricide exposure to non-target species and minimal environmental contamination [78]. Bait tube technologies and modified live traps with topical acaricide delivery systems have already proven their efficacy in reducing tick numbers on rodents and their associated habitat, in several states in the USA [78–80]. Nevertheless, the main disadvantages of these methods are the high cost and labour intensiveness. Apart from rodents, urban hedgehogs seem to play a vital role in the ecology of ticks in Cluj-Napoca, thus modified versions of such traps targeting these “tick taxies” could be tested to reduce tick populations in the urban scenery.

## Conclusions

The outcome of this study performed in areas not previously subjected to epidemiological investigation shows that ticks are present in a variety of green habitat types within the urban/peri-urban scenery assessed in Cluj-Napoca and that all sites surveyed supported ticks. *Ixodes ricinus* was the dominant tick species both as free stages as well as engorged on wildlife hosts. Several abiotic and biotic factors shape the ecology of ticks in the city of Cluj-Napoca, with climate and the local presence of suitable hosts being the most important.

## Supplementary information

**Additional file 1: Table S1.** The schedule used for the flagging, micromammal trapping, and bird and hedgehog sampling campaigns in Cluj-Napoca during 2018. Numbers indicate weeks of the year. The timeline of the protocol used for the flagging, sampling, and trapping campaigns performed in the seven recreational locations in Cluj-Napoca during 2018 is provided.

**Additional file 2: Table S2.** Vegetation cover of individual locations of urban and peri-urban green areas in Cluj-Napoca. The number of vegetation sampling plots and percentages of grass, shrub and arboreal cover in each of the seven locations assessed in Cluj-Napoca during 2018 are provided.

## Data Availability

All data generated during this study are included in this published article and its additional files.

## Abbreviations

*s.l*: *sensu lato*
USAMV Campus: University of Agricultural Sciences and Veterinary Medicine
AI: abundance indices
AIL: the abundance index of larvae of *I. ricinus*
AIN: the abundance index of nymphs of *I. ricinus*
AIA: the abundance index of adults of *I. ricinus*
AIT: the total abundance index of *I. ricinus*
AIG: the total abundance index of ticks regardless of species and stage
TR: the number of ticks collected in a certain location
*a*: the surface of the sampled area in square meters
*P*: significance level
CI: confidence interval
*χ*^2^: Chi-square test
rho: Spearman’s rank correlation coefficient
RH: relative humidity
*df*: degrees of freedom
TBPs: ticks-borne pathogens

## References

[1] de la Fuente J, Estrada-Peña A, Venzal JM, Kocan KM, Sonenshine DE (2008). Overview: ticks as vectors of pathogens that cause disease in humans and animals. Front Biosci.

[2] Sonenshine DE, Roe RM (2013). Biology of ticks.

[3] Karesh WB, Dobson A, Lloyd-Smith JO, Lubroth J, Dixon MA, Bennett M (2012). Ecology of zoonoses: natural and unnatural histories. Lancet.

[4] Bradley CA, Altizer S (2007). Urbanization and the ecology of wildlife diseases. Trends Ecol Evol.

[5] Estrada-Peña A, de la Fuente J (2014). The ecology of ticks and epidemiology of tick-borne viral diseases. Antiviral Res.

[6] Uspensky I (2014). Tick pests and vectors (Acari: Ixodoidea) in European towns: introduction, persistence and management. Ticks Tick Borne Dis.

[7] Vandecasteele I, Baranzelli C, Siragusa A, Aurambout JP, Alberti V, Alonso Raposo M, et al. The future of cities. In: Opportunities, challenges and the way forward. Publications Office of the European Union. 2019. https://ec.europa.eu/jrc/en/facts4eufuture/future-of-cities. Accessed 6 Jul 2020.

[8] Ginsberg HS, Faulde MK, Bonnefoy X, Kampen H, Sweeney K (2008). Ticks. Public health significance of urban pests.

[9] Rizzoli A, Silaghi C, Obiegala A, Rudolf I, Hubálek Z, Földvári G (2014). *Ixodes ricinus* and its transmitted pathogens in urban and peri-urban areas in Europe: new hazards and relevance for public health. Front Public Health.

[10] Pfäffle M, Littwin N, Muders SV, Petney TN (2013). The ecology of tick-borne diseases. Int J Parasitol.

[11] Heyman P, Cochez C, Hofhuis A, Van Der Giessen J, Sprong H, Porter SR (2010). A clear and present danger: tick-borne diseases in Europe. Expert Rev Anti Infect Ther.

[12] Léger E, Vourc’h G, Vial L, Chevillon C, McCoy KD (2013). Changing distributions of ticks: causes and consequences. Exp Appl Acarol..

[13] Medlock JM, Hansford KM, Bormane A, Derdakova M, Estrada-Peña A, George JC (2013). Driving forces for changes in geographical distribution of *Ixodes ricinus* ticks in Europe. Parasit Vectors.

[14] Niemelä J, Breuste JH, Guntenspergen G, McIntyre NE, Elmqvist T, James P (2011). Urban ecology: patterns, processes, and applications.

[15] Santos AS, de Bruin A, Veloso AR, Marques C, da Fonseca IP, de Sousa R (2018). Detection of Anaplasma phagocytophilum, Candidatus *Neoehrlichia* sp, *Coxiella burnetii* and *Rickettsia* spp in questing ticks from a recreational park, Portugal. Ticks Tick Borne Dis..

[16] Szekeres S, Lügner J, Fingerle V, Margos G, Földvári G (2017). Prevalence of *Borrelia miyamotoi* and *Borrelia burgdorferi sensu lato* in questing ticks from a recreational coniferous forest of East Saxony. Germany Ticks Tick Borne Dis.

[17] Vârban R, Vârban D, Stoie A (2010). The preservation of nature and biodiversity in the county of Cluj. Ann Univ Oradea Environ Protection.

[18] Raileanu C, Moutailler S, Pavel I, Porea D, Mihalca AD, Savuta G (2017). *Borrelia* diversity and co-infection with other tick borne pathogens in ticks. Front Cell Infect Microbiol.

[19] Raileanu C, Moutailler S, Porea D, Oslobanu L, Anita D, Anita A (2018). Molecular evidence of *Rickettsia* spp, *Anaplasma phagocytophilum*, and “*Candidatus* Neoehrlichia mikurensis” in ticks from natural and urban habitats in eastern Romania. Vector Borne Zoonotic Dis.

[20] Andersson MO, Tolf C, Tamba P, Stefanache M, Radbea G, Rubel F (2017). *Babesia*, *Theileria*, and *Hepatozoon* species in ticks infesting animal hosts in Romania. Parasitol Res.

[21] Andersson MO, Tolf C, Tamba P, Stefanache M, Radbea G, Frangoulidis D (2018). Molecular survey of neglected bacterial pathogens reveals an abundant diversity of species and genotypes in ticks collected from animal hosts across Romania. Parasit Vectors.

[22] Andersson MO, Zaghdoudi-Allan N, Tamba P, Stefanache M, Chitimia L (2014). Co-infection with ‘*Candidatus* Neoehrlichia mikurensis’ and *Borrelia afzelii* in an *Ixodes ricinus* tick that has bitten a human in Romania. Ticks Tick Borne Dis.

[23] Briciu VT, Meyer F, Sebah D, Tatulescu DF, Coroiu G, Lupșe M (2014). Real-time PCR-based identification of *Borrelia burgdorferi sensu lato* species in ticks collected from humans in Romania. Ticks Tick Borne Dis.

[24] Matei IA, Kalmár Z, Lupşe M, D’Amico G, Ionică AM, Dumitrache MO (2017). The risk of exposure to rickettsial infections and human granulocytic anaplasmosis associated with *Ixodes ricinus* tick bites in humans in Romania: a multiannual study. Ticks Tick Borne Dis.

[25] Andersson MO, Marga G, Banu T, Dobler G, Chitimia-Dobler L (2018). Tick-borne pathogens in tick species infesting humans in Sibiu County, central Romania. Parasitol Res.

[26] Pavel I, Miron L, Raileanu C, Macovei II, Tronciu C, Acatrinei DM (2014). Seasonal dynamics of ixodid ticks in Iași urban area. Lucr St USAMV Iaşi.

[27] Cluj County Directorate of Statistics. Population of Cluj county on January 1st, 2019 by address. 2019. https://cluj.insse.ro/populatia-la-1-iulie-2017-dupa-domiciliu/. Accessed 21 Jun 2019 **(in Romanian)**.

[28] National Institute of Statistics. Romania’s population by locality on January 1, 2016; 2016. https://web.archive.org/web/20171027131447/; https://www.insse.ro/cms/ro/content/popula%C5%A3ia-rom%C3%A2niei-pe-localitati-la-1-ianuarie-2016. Accessed 21 Jun 2019 **(in Romanian)**.

[29] Meteoblue Weather. Cluj-Napoca Weather Archive; 2019. https://www.meteoblue.com/ro/vreme/prognoza/archive/clujnapoca_rom%c3%a2nia_681290?fcstlength=1m&year=2018&month=1. Accessed 14 Jun 2019 **(in Romanian)**.

[30] West G, Heard D, Caulkett N (2007). Zoo Animal and wildlife immobilization and anesthesia.

[31] Mullineaux E, Best R, Cooper JE (2003). Manual of wildlife casualities.

[32] Svensson L (1992). Identification guide to European passerines.

[33] Popescu A, Murariu D. Romania’s Fauna, Mammalia, Rodentia. 16: 2Bucharest: The Publishing House Of The Romanian Academy; 2001 **(in Romanian)**.

[34] Estrada-Peña A, Mihalca AD, Petney TN (2018). Ticks of Europe and North Africa: a guide to species identification.

[35] Aureli S, Galuppi R, Ostanello F, Foley JE, Bonoli C, Rejmanek D (2015). Abundance of questing ticks and molecular evidence for pathogens in ticks in three parks of Emilia-Romagna region of northern Italy. Ann Agric Environ Med.

[36] Hansford KM, Fonville M, Gillingham EL, Coipan EC, Pietzsch ME, Krawczyk AI (2017). Ticks and *Borrelia* in urban and peri-urban green space habitats in a city in southern England. Ticks Tick Borne Dis.

[37] Mihalca AD, Dumitrache MO, Sándor AD, Magdaş C, Oltean M, Györke A (2012). Tick parasites of rodents in Romania: host preferences, community structure and geographical distribution. Parasit Vectors.

[38] Domșa C, Mihalca AD, Sándor AD (2018). Modeling the distribution of *Ixodes ricinus* in Romania. North West J Zool.

[39] Oechslin CP, Heutschi D, Lenz N, Tischhauser W, Péter O, Rais O (2017). Prevalence of tick-borne pathogens in questing *Ixodes ricinus* ticks in urban and suburban areas of Switzerland. Parasit Vectors.

[40] Kubiak K, Dziekonska-Rynko J (2006). Seasonal activity of the common European tick, *Ixodes ricinus* (*Linnaeus*, 1758), in the forested areas of the city of Olsztyn and its sorroundings. Wiad Parazytol.

[41] Briciu VT, Titilincu A, Țățulescu DF, Cârstina D, Lefkaditis M, Mihalca AD (2011). First survey on hard ticks (*Ixodidae*) collected from humans in Romania: possible risks for tick-borne diseases. Exp Appl Acarol.

[42] Nosek J (1971). The ecology, bionomics and behaviour of *Haemaphysalis* (Haemaphysalis) *concinna* tick. Zeitschr Parasitenkde.

[43] Palomar AM, Portillo A, Santibáñez P, Mazuelas D, Roncero L, García-Álvarez L (2015). Detection of tick-borne *Anaplasma bovis*, *Anaplasma phagocytophilum* and *Anaplasma centrale* in Spain. Med Vet Entomol.

[44] Wilson ML, Sonenshine DE, Mater TN (1994). Population ecology of tick vectors: interaction, measurement and analysis. Ecological dynamics of tick-borne zoonoses.

[45] Daniel M, Malý M, Danielová V, Kříž B, Nuttall P (2015). Abiotic predictors and annual seasonal dynamics of *Ixodes ricinus*, the major disease vector of central Europe. Parasit Vectors.

[46] Kowalec M, Szewczyk T, Welc-Falęciak R, Siński E, Karbowiak G, Bajer A (2017). Ticks and the city-are there any differences between city parks and natural forests in terms of tick abundance and prevalence of spirochaetes?. Parasit Vectors.

[47] Tews J, Brose U, Grimm V, Tielbörger K, Wichmann MC, Schwager M (2004). Animal species diversity driven by habitat heterogeneity/diversity: the importance of keystone structures. J Biogeogr.

[48] Lowry H, Lill A, Wong BB (2013). Behavioural responses of wildlife to urban environments. Biol Rev.

[49] Gilbert L, Maffey GL, Ramsay SL, Hester AJ (2012). The effect of deer management on the abundance of *Ixodes ricinus* in Scotland. Ecol Appl.

[50] Gern L, Rouvinez E, Toutoungi LN, Godfroid E (1997). Transmission cycles of *Borrelia burgdorferi sensu lato* involving *Ixodes ricinus* and/or *I hexagonus* ticks and the European hedgehog, *Erinaceus europaeus*, in suburban and urban areas in Switzerland. Folia Parasitol.

[51] Földvári G, Rigó K, Jablonszky M, Biró N, Majoros G, Molnár V (2011). Ticks and the city: ectoparasites of the northern white-breasted hedgehog (*Erinaceus roumanicus*) in an urban park. Ticks Tick Borne Dis.

[52] Medlock JM, Hansford KM, Vaux AGC, Cull B, Pietzsch ME, Gillingham EL (2018). Has the red sheep tick, *Haemaphysalis punctata*, recently expanded its range in England?. Med Vet Entomol.

[53] Dobson AD, Taylor JL, Randolph SE (2011). Tick (*Ixodes ricinus*) abundance and seasonality at recreational sites in the UK: hazards in relation to fine-scale habitat types revealed by complementary sampling methods. Ticks Tick Borne Dis.

[54] Nuttall PA, Labuda M, Sonenshine DE, Mather TN (1994). Tick-borne encephalitis subgroup. Ecological dynamics of tick-borne zoonoses.

[55] Kurtenbach K, Hanincová K, Tsao JI, Margos G, Fish D, Ogden NH (2006). Fundamental processes in the evolutionary ecology of Lyme borreliosis. Nat Rev Microbiol.

[56] Hamer SA, Hickling GJ, Sidge JL, Walker ED, Tsao JI (2012). Synchronous phenology of juvenile *Ixodes scapularis*, vertebrate host relationships, and associated patterns of *Borrelia burgdorferi* ribotypes in the midwestern United States. Ticks Tick Borne Dis.

[57] Hartemink N, Van Vliet A, Sprong H, Jacobs F, Garcia-Martí I, Zurita-Milla R (2019). Temporal-spatial variation in questing tick activity in the Netherlands: the effect of climatic and habitat factors. Vector Borne Zoonotic Dis.

[58] Balashov YS. A translation of bloodsucking ticks (Ixodoidea)-vectors of disease in man and animals. In: Strekalovsky OG, Hoogstraal H, Tatchell RJ, editors. Miscellaneous publications of the entomological society of America. College Park: Entomological Society of America; 1972.

[59] Boehnke D, Gebhardt R, Petney T, Norra S (2017). On the complexity of measuring forests microclimate and interpreting its relevance in habitat ecology: the example of *Ixodes ricinus* ticks. Parasit Vectors.

[60] Anderson JF, Magnarelli LA (2008). Biology of ticks. Infect Dis Clin North Am.

[61] Dziemian S, Sikora B, Piłacińska B, Michalik J, Zwolak R (2015). Ectoparasite loads in sympatric urban populations of the northern white-breasted and the European hedgehog. Parasitol Res.

[62] Dziemian S, Michalik J, Piłacińska B, Bialik S, Sikora B, Zwolak R (2014). Infestation of urban populations of the northern white-breasted hedgehog, *Erinaceus roumanicus*, by *Ixodes* spp ticks in Poland. Med Vet Entomol.

[63] D’Amico G, Dumitrache MO, Matei IA, Ionică AM, Gherman CM, Sándor AD (2017). Ixodid ticks parasitizing wild carnivores in Romania. Exp Appl Acarol.

[64] Sándor AD, D’Amico G, Gherman CM, Dumitrache MO, Domșa C, Mihalca AD (2017). Mesocarnivores and macroparasites: altitude and land use predict the ticks occurring on red foxes (*Vulpes vulpes*). Parasit Vectors.

[65] Steere AC, Strle F, Wormser GP, Hu LT, Branda JA, Hovius JW (2016). Lyme borreliosis Nat Rev Dis Primers.

[66] Hubálek, Z. Epidemiology of Lyme borreliosis. In: Lipsker D, Jaulhac B, editors.Lyme borreliosis. Biological and Clinical Aspects. Current Problems in Dermatology. Basel, Karger; 2009;37:31–50. 10.1159/000213069 19367096

[67] Hamer SA, Goldberg TL, Kitron UD, Brawn JD, Anderson TK, Loss SR (2012). Wild birds and urban ecology of ticks and tick-borne pathogens, Chicago, Illinois, USA, 2005–2010. Emerg Infect Dis.

[68] Sándor AD, Mărcuţan DI, D’Amico G, Gherman CM, Dumitrache MO, Mihalca AD (2014). Do the ticks of birds at an important migratory hotspot reflect the seasonal dynamics of *Ixodes ricinus* at the migration initiation site? A case study in the Danube Delta. PLoS ONE.

[69] Klaus C, Gethmann J, Hoffmann B, Ziegler U, Heller M, Beer M (2016). Tick infestation in birds and prevalence of pathogens in ticks collected from different places in Germany. Parasitol Res.

[70] Sándor AD, Kalmár Z, Matei IA, Ionică AM, Mărcuţan DI (2017). Urban breeding corvids as disseminators of ticks and emerging tick-borne pathogens. Vector Borne Zoonotic Dis.

[71] Luniak M. Synurbization-adaptation of animal wildlife to urban development. In: Procceedings 4th International Urban Wildlife Symposium, Tucson, USA; 2004. p. 50–5.

[72] Maaz D, Krücken J, Blümke J, Richter D, McKay-Demeler J, Matuschka FR (2018). Factors associated with diversity, quantity and zoonotic potential of ectoparasites on urban mice and voles. PLoS ONE.

[73] Mihalca AD, Gherman CM, Magdaş C, Dumitrache MO, Györke A, Sándor AD (2012). *Ixodes ricinus* is the dominant questing tick in forest habitats in Romania: the results from a countrywide dragging campaign. Exp Appl Acarol.

[74] de Mendonça PG, Benedek AM, Jurčovičová M (2011). Molecular screening of European wild rodents for tick-borne encephalitis virus. Acta Zool Bulg.

[75] Mihalca AD, Sándor AD (2013). The role of rodents in the ecology of *Ixodes ricinus* and associated pathogens in central and eastern Europe. Front Cell Infect Microbiol.

[76] Haase D, Larondelle N, Andersson E, Artmann M, Borgström S, Breuste J (2014). A quantitative review of urban ecosystem service assessments: concepts, models, and implementation. Ambio.

[77] Medlock JM, Shuttleworth H, Copley V, Hansford KM, Leach S (2012). Woodland biodiversity management as a tool for reducing human exposure to *Ixodes ricinus* ticks: a preliminary study in an English woodland. J Vector Ecol.

[78] Dolan MC, Maupin GO, Schneider BS, Denatale C, Hamon N, Cole C (2004). Control of immature *Ixodes scapularis* (Acari: Ixodidae) on rodent reservoirs of *Borrelia burgdorferi* in a residential community of southeastern Connecticut. J Med Entomol.

[79] Gage KL, Maupin GO, Montenieri J, Piesman J, Dolan M, Panella NA (1997). Flea (Siphonaptera: Ceratophyllidae, Hystrichopsyllidae) and tick (Acarina: Ixodidae) control on wood rats using host-targeted liquid permethrin in bait tubes. J Med Entomol.

[80] Lane R, Casher L, Peavey C, Piesman J (1998). A better tick-control trap: modified bait tube controls disease-carrying ticks and fleas. Calif Agric.

