# Supplementary material for: Impact of abiotic factors, habitat type and urban wildlife on the ecology of hard ticks (Acari: Ixodidae) in urban and peri-urban habitats
Source: Parasit Vectors. 2020 Sep 18;13:476. doi: 10.1186/s13071-020-04352-3 (PMC7501622; doi:10.1186/s13071-020-04352-3)
Supplement: Supplementary file 2 — Additional file 2: Table S2. Vegetation cover of individual locations of urban and peri-urban green areas in Cluj-Napoca. The number of vegetation sampling plots and percentages of grass, shrub and arboreal cover in each of the seven locations assessed in Cluj-Napoca during 2018 are provided. [file 13071_2020_4352_MOESM2_ESM.docx]

**Additional file 2: Table S2.** Vegetation cover of individual locations of urban and peri-urban green areas in Cluj-Napoca. The number of vegetation sampling plots and percentages of grass, shrub and arboreal cover in each of the seven locations assessed in Cluj-Napoca during 2018 are provided.

| Site no. | Location | No. of vegetation sampling plots | Grass cover (%) | Shrub cover (%) | Arboreal cover (%) |
| --- | --- | --- | --- | --- | --- |
| 1 | USAMV Campus | 14 | 77.14 (±0.115) | 21.43 (±0.128) | 35.71 (±0.119) |
| 2 | Mănăștur Cemetery | 20 | 66.75 (±0.13) | 11.75 (±0.069) | 1.70 (±0.014) |
| 3 | Iuliu Hațieganu Park | 16 | 71.88 (±0.128) | 7.81 (±0.032) | 30.00 (±0.08) |
| 4 | Alexandru Borza Botanical Garden | 16 | 78.13 (±0.026) | 20.31 (±0.036) | 19.38 (±0.056) |
| 5 | Hoia forest | 14 | 61.43 (±0.193) | 22.86 (±0.129) | 21.43 (±0.156) |
| 6 | Făget forest | 12 | 57.50 (±0.173) | 26.67 (±0.091) | 44.17 (±0.255) |
| 7 | Private central garden | 4 | 70.00 (±0.277) | 15.00 (±0.098) | 7.50 (±0.049) |
